# The haemosporidian parasites of bats with description of *Sprattiella alecto* gen. nov., sp. nov.

**DOI:** 10.1051/parasite/2012192137

**Published:** 2012-05-15

**Authors:** I. Landau, J.M. Chavatte, G. Karadjian, A. Chabaud, I. Beveridge

**Affiliations:** 1 Parasitologie comparée et Modèles expérimentaux, USM 307, Muséum National d’Histoire Naturelle 61, rue Buffon CP 52 75231 Paris Cedex 05 France; 2 National Public Health Laboratory – Malaria Reference Centre, Ministry of Health, 9 Hospital Drive, Block C, #04-01, SingHealth Research Facilities Singapore 169612; 3 Department of Veterinary Science, University of Melbourne Parkville, Victoria Australia

**Keywords:** *Sprattiella* gen. nov., *alecto* sp. nov., Haemoproteidae, Haemosporidia, Chiroptera, *Sprattiella* gen. nov., *alecto* sp. nov., hémoprotéidés, hémosporidies, chiroptères

## Abstract

Four species of Haemoproteidae were found in *Pteropus alecto* Temminck, 1837 in Queensland, Australia: i) *Johnsprentia copemani*, Landau *et al.*, 2012; ii) *Sprattiella alecto* gen. nov., sp. nov., characterised by schizonts in the renal vessels; iii) *Hepatocystis levinei*, Landau *et al.*, 1985, originally described from *Pteropus poliocephalus* Temminck, 1825 and, experimentally from *Culicoides nubeculosus* and found in this new host and for which features of the hepatic schizonts are reported; iv) gametocytes of *Hepatocystis* sp. which are illustrated but cannot be assigned to a known species. A tentative interpretation of phylogenetic characters of haemosporidians of bats is provided from the morphology of the gametocytes and localisation of the tissue stages with respect to recent data on the phylogeny of bats.

## Abstract (fr)

Quatre espèces d’hémoproteidés parasitent les *Pteropus alecto* Temminck, 1837 du Queensland : i) *Johnsprentia copemani*, Landau *et al.*, 2012 ; ii) *Sprattiella alecto* gen. nov., sp. nov., caractérisée par des schizontes siégeant dans la lumière des vaisseaux du rein ; iii) *Hepatocystis levinei*, Landau *et al.*, 1985, décrite chez *Pteropus poliocephalus* et, expérimentalement, chez *Culicoides nubeculosus* ; l’espèce est retrouvée chez ce nouvel hôte et des compléments à la description des schizontes hépatiques sont apportés ; iv) *Hepatocystis* sp., dont les gamétocytes sont figurés mais n’ont pas pu être rattachés à une espèce particulière. Une tentative d’interprétation des caractères évolutifs des Hémosporidies de Chiroptères en fonction de la morphologie des gamétocytes, de la localisation des stades tissulaires et des données récentes sur la phylogénie des Chauve-souris, est ici présentée.

## Introduction

Gametocytes of haemoproteids have frequently been reported in flying foxes (Pteropodidae). In the absence of information on the localization of their schizonts, they have been placed in the genus *Hepatocystis*. This genus was defined by (Garnahm, 1966) as having “megaloschizonts” in the liver and gametogony stages in the blood. In reality, the haemoproteids of bats are highly ivvariable with respect to such characters and their representatives in *Pteropus alecto* Temminck, 1837 from Queensland, provide an excellent example of this diversity. They are:

- *Hepatocystis levinei* described by Landau *et al.* (1985) from *Pteropus poliocephalus* Temminck, 1825. The life cycle was completed using *Culicoides nubeculosus* under laboratory conditions (Landau *et al.*, 1985). This species was found in four of 11 *P. alecto* examined from Townsville, Queensland, Australia, and we here provide additional data on the hepatic schizonts;
- *Johnsprentia copemani* described by Landau *et al.* (2012) in *P. alecto* from Queensland. Co-infection with other haemoproteids was reported in the same paper;
- *Hepatocystis* sp. represented by gametocytes in the blood of four of the 11 bats examined in the present study, but no schizonts were found which could be attributed to this species;
- *Sprattiella alecto* gen. nov., sp. nov., herein described, gametocytes in the blood of six of 11 bats examined and characteristic schizonts in vessels of the kidney in one of the bats examined.

Recent advances of phylogeny studies of the Pteropodidae based on molecular methods prompted us to reevaluate the morphological features of haemoproteids of bats and to compare them with the molecular data currently available for the hosts.

## Materials and Methods

The methods used were essentially the same as those used in the description of *Johnsprentia copemani* by Landau *et al.* (2012).

Eleven *P. alecto*, captured at Townsville using a mist net and exhibiting a parasitaemia with Haemoproteidae, were transported to Paris, to the Muséum National d’Histoire Naturelle, shortly after their capture, arriving on the 15.12.1978 and the 07.06.1979, respectively. Blood samples from each animal were collected by pricking the radial vein and the blood was smeared onto a slide, air dried, fixed with absolute methanol and stained using Giemsa stain (8 % in phosphate buffer, pH 7.4). The bats were examined over a period of several months. At autopsy, internal organs were fixed in Carnoy’s fluid and serial sections of each organ were stained by the giemsa-colophonium method (Bray & Garnham, 1962; Garnahm, 1966) and examined for tissue stages of the parasites. Type material has been deposited in the Muséum National d’Histoire Naturelle (MNHN), Paris.

Bat number 850-932PX in which we found renal schizonts was autopsied on the 03-01-1979, after 19 days in captivity.

## Systematics

- Phylum: Apicomplexa (Sporozoa).
- Class: Coccidea.
- Order: Haemosporida.
- Family: Haemoproteidae.
- Genus: *Sprattiella* gen. nov.
- Etymology: named after Dr David Spratt.
- Definition: Haemoproteidae of Pteropodidae with relatively small elongated schizonts, not producing colloid, inside a hypertrophied bi-nucleated host cell, free in the large vessels of the kidneys; gametocytes in erythrocytes.
- Type species: *Sprattiella alecto* sp. nov.
- Etymology: named after the host specific name.

## Descriptions

### *Sprattiella alecto* sp. nov. (figs 1-8, 11–24)

Holotype material: schizont in section of kidney from *P. alecto* (850-932PX), MNHN P2 XXXI 10 (Fig. 7). Paratypes: blood smears and sections of schizonts in kidney, deposited in MNHN.

**Figs 1–8.**
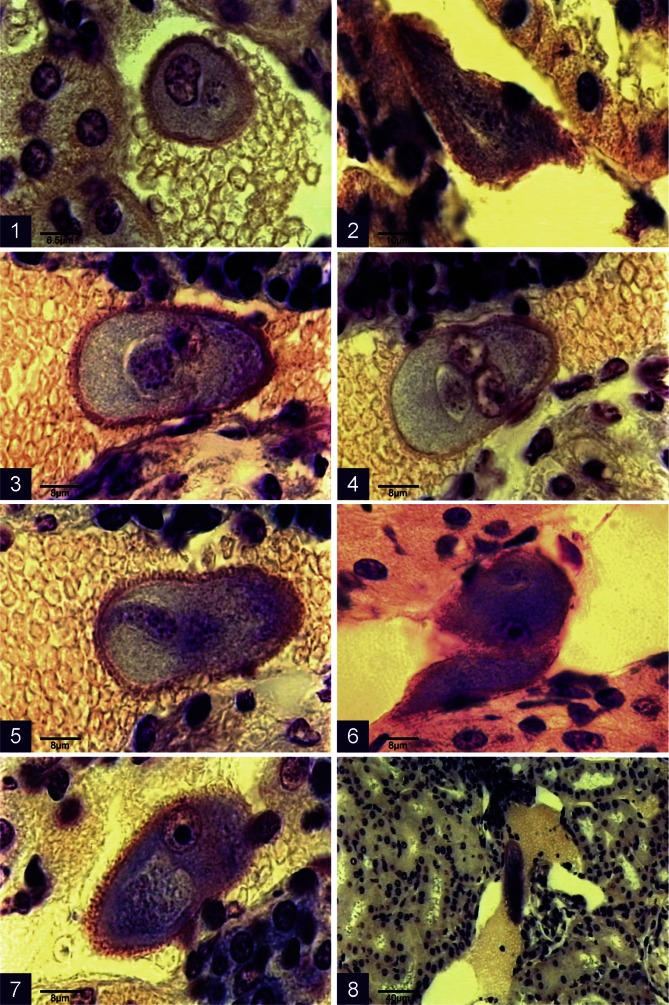
Microphotographs of schizonts of *Sprattiella alecto* in the blood vessels of kidney sections from *Pteropus alecto*

**Figs 9–10 & 11–30.**
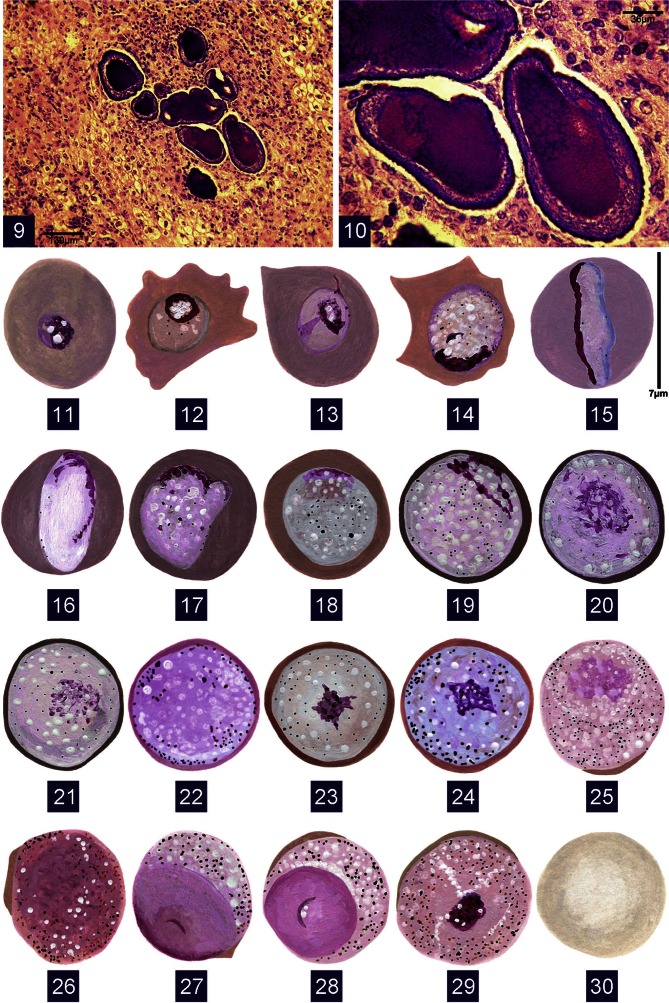
Fig. 9: microphotograh of schizonts of *Hepatocystis levinei* in liver sections from *Pteropus alecto*; Fig. 10: detail of Fig. 9; Figs 11-30: giemsa stain; Figs 11-24: drawings of gametocytes of *Sprattiella alecto*; Fig. 11: very young trophozoite; Figs 12-18: young trophozoites; Figs 18-21: immature microgametocytes; Fig. 22: mature microgametocyte; Figs 23-24: macrogametocytes; Figs 25-28: microgametocytes of *Hepatocystis* sp.; Fig. 29: Microgametocyte “en cocarde” of *Hepatocystis levinei*; Fig. 30: uninfected RBC

• Renal schizonts

Found in the lumen of renal veins, inside hypertrophied cells invariably with two nuclei (Fig. 4); nuclei also hypertrophied and modified. Host cell nuclei ellipsoidal, up to 10.5 μm with chromatin concentrated around the periphery and a large nucleolus. Host cell surrounded by fibrous, bright pink substance, up to 2 μm in thickness.

Parasitized cells free in vessels in most sections (Fig. 1), but in some serial sections, apparently attached to the walls of veins (Fig. 2) or wedged by a shrinkage of vessels (Figs 5, 6); round or oval depending on the angle of the section, sometimes deformed when trapped in shrunken vessel (Fig. 6).

The smallest schizont observed was in an enlarged, binucleate rounded cell measuring 16.5 μm, with a dense basophilic cytoplasm. The cell was surrounded by an irregular, pink, tufted layer. The schizont, by contrast, was small, 4 μm in diameter, containing a dozen small nuclei with little, pale cytoplasm. The schizont develops as cordons, at first straight with small well-spaced nuclei (Figs 2, 5), then reflected upon themselves (Figs 7, 8). The enlarged host cell (Fig. 3) is pyriform with a uniformly granular, grey cytoplasm, containing distinctive nuclei: oval 2-2.5 μm long with a clear centre and a thin membrane with masses of chromatin attached to its inner surface. These nuclei subsequently fragment and the oldest schizont observed (Fig. 8) measured 83 × 21 μm with elongated masses within the host cell with a basophilic cytoplasm and numerous nuclei.

• Gametocytes (Figs 11-24)

In the serial blood films examined, gametocytes were more numerous in the middle and terminal thirds of the films than in other regions. They lodge in red blood cells (RBC) and from the young trophozoite stage undergo significant transformation. The RBC is generally rounded but may become deformed with young parasites either irregularly (Fig. 12) or pyriform (Fig. 13). The parasite is either a clear reddish-brown, or more commonly darker, reddish or brick-red. The gametocytes increase in size slightly (7 μm); the intact host cell is still visible surrounding the gametocyte which against the dark background appears clear, measuring between 6 and 7 μm.

The microgametocytes (Figs 19-22) are pale pink or pink with several vacuoles and a more or less elongated zone of chromatin forming small, loosely attached masses. The macrogametocyte (Figs 23, 24) is pale blue to almost white against the darkly staining red blood cell background; its nucleus is smaller, and more dense than that of the microgametocyte. Pigment in both micro- and macrogametocytes is fine, scattered and more abundant towards the periphery. The microgametocytes of *Sprattiella* belong within the *vivax* group (*vide infra*).

• Relationship between the gametocytes of *Sprattiella* and renal schizonts

In multiple infections it is often difficult to associate gametocytes with their corresponding schizonts. In the blood of bat 850-932PX, we observed the gametocytes of four species of Haemoproteidae. We already know the gametocytes of three species: *Johnsprentia copemani*, *Hepatocystis levinei* and *Hepatocystis* sp. It is therefore logical to associate the renal schizonts with the distinctive gametocytes described here. Consequently, the holotype of *Sprattiella alecto* has been designated as the renal schizont and the name is therefore attached to this stage.

• Differential diagnosis

The gametocytes of *Sprattiella* differ from those of the other three known species present in *P. alecto* due to their striking effect on the host cell and the following morphological features: the nucleus of the microgametocyte is not peripheral in contrast to the other species; in addition, the nucleus of *Sprattiella* is diffuse in contrast to that of *Johnsprentia* which is in the form of a crescent and that of *H. levinei* which has a condensed center surrounded by a clear space.

*Sprattiella alecto* is also compared with the other two haemoproteids in which the schizont and its hypertrophied host cell are found in the lumen of medium to large blood vessels.

 i) The schizonts of *Dionisia bunoi* are found in the blood vessels of the liver of the microchiropteran *Hipposideros cyclops* (Landau, Chabaud, *et al*., 1980b). They exhibit several characters in common with the present parasite: an intravascular localisation, host cell and nucleus hypertrophied and the presence of a hirsute pink substance around the periphery. However, they are exclusively hepatic and not renal. The schizont of *Dionisia* is anchored by prolongations from the host cell to the hepatic capillaries of the surrounding tissue while that of *Sprattiella* is free. The schizont of *Dionisia* forms a solid rounded mass while that of *Sprattiella* consists of elongated cords. The unique host nucleus of *Dionisia* becomes very large and highly modified while those of *Sprattiella* only undergo a moderate increase in volume. Finally and of most significance, the microgametocytes of *Dionisia* are of the “*falciparum*” type and the microgametocytes of the “*malariae*” type; those from *Pteropus* – host are of the “*vivax*” type. ii) The schizonts of *Polychromophilus* are found in the lumen of vessel, primarily in the lung, but also in other organs (liver, kidney, adrenals) (Landau, Rosin, *et al*., 1980a); the host cell is only visible in very young schizonts and is reduced in older forms to a slightly hypertrophied nucleus and a peripheral band of homogeneous, bright red ‘colloid’, different to the tufted band in *Sprattiella*. The gametocytes of *Polychromophilus* are of the “*malariae*” type.

### Hepatocystis levinei

• Additions to the description of the hepatic schizonts Schizonts and gametocytes belonging to *H. levinei* were found in the blood and in liver sections of several *P. alecto*, having first been reported from *Pteropus poliocephalus* by Landau *et al.* (1985). We had not, at the time, observed all of the hepatic schizogonic stages, in particular the intermediate stage between the young, stellate schizont lacking colloid and the mature schizont. Here, we illustrate (Fig. 29) a characteristic microgametocyte with the condensed center and a mature schizont in a section through several lobes, each lobe comprising a peripheral zone with numerous small nuclei and a central zone filled with purple colloid. In the section in which the parasite is largest, the parasite and the surrounding zone of inflammation measure 1,700 × 2,700 μm; the parasite itself measures 630 × 370 μm.

### *Hepatocystis* sp.

The microgametocytes of this species are of the “*vivax*” type with a diffuse nucleus (Figs 25-28). Macrogametocytes could not be distinguished from those of other species.

## Discussion

### The different types of haemosporidian gametocytes in mammals

The morphology of haemosporidian gametocytes in mammals is generally considered to be of limited taxonomic interest. By contrast, we consider that because of their stability and their archaic nature (they represent a fundamental primitive stage in the cycle of all coccidiomorphs), they are probably of considerable importance from a phyletic and systematic perspective.

Schizogony, be it haematogenous or in tissues, is a superimposed character with numerous morphological variants including convergences between distant species. Landau *et al.* (1976a), based on the morphology of the gametocytes of mammalian haemosporidians, divided them into three types (Table I) which were designated by the name of the best known species in each group (“*falciparum*”, considered the most archaic, then “*malariae*” and finally “*vivax*”).

**Table I.** Morphological characteristics of microgametocytes of mammalian haemosporidians (Landau *et al*., 1976, modified).

	Type of microgametocyte		
Morphological characteristic	*falciparum*	*malariae*	*vivax*
Filling completely or almost the host RBC	no	no	yes
Form	ellipsoidal or elongate	rounded	rounded
Eccentric nucleus	no	no	yes
Distinct limit between nucleus and cytoplasm	no	yes	yes
Accessory chromatin dot	frequent	frequent	absent
Distinct border between nucleus and pigmented zone	no	no	yes
Pigment granules	few and coarse	Few and coarse	fine and abundant
Distribution of pigment	irregular, often grouped	irregular, often grouped	dispersed

The classification is based on the morphology of the microgametocytes which are the most characteristic and most readily recognizable features of *Plasmodium*.

The principal morphological characters of the three types are summarised in Table I and their distribution in different types of bats is presented in Table II. In addition, a dichotomous key for the identification of the genera of haemoproteids in bats is presented in Table III.

**Table II.** Haemosporidia of Chiroptera.

	Host	Genus	Species	Authors	Gametocyte type	Schizont location
Nycteridae	*Nycteris arge*	*Nycteria*	*erardi*	Rosin , Landau *et al*., 1978	M	HEP
	*Nycteris capensis*	*Nycteria*	*medusiformis*	Garnahm & Heisch, 1953	M	HEP
	*Nycteris nana*	*Nycteria*	*houini*	Rosin , Landau *et al*., 1978	M	HEP
	*Nycteris thebaica*	*Nycteria*	*medusiformis*	Garnahm & Heisch, 1953	M	HEP

Vespertilionidae	*Eptesicus fuscus*	*Bioccala*	*deanei*	(Garnahm Lainson *et al*., 1971,)**	F	RES
	*Vespertilio murinus*	*Bioccala*	*murinus*	(Dionisi, 1899)	F	RES

Miniopteridae	*Miniopterus minor*	*Polychromophilus*	*adami*	Landau, Rosin, *et al*., 1980a	M	RES
	*Miniopterus minor*	*Bioccala*	*murinus*	(Dionisi, 1899)	F	RES
	*Miniopterus schreibersi*	*Polychromophilus*	*melaniferus*	(Dionisi, 1899)	M	RES
	*Miniopterus schreibersi*	*Polychromophilus*	*corradettii*	Landau, Rosin, et al., 1980a	M	RES
	*Miniopterus schreibersi*	*Bioccala*	*murinus*	(Dionisi, 1899)	F	RES
	*Myotis myotis*	*Bioccala*	*murinus*	(Dionisi, 1899)	F	RES
	*Myotis natterei*	*Bioccala*	*murinus*	(Dionisi, 1899)	F	RES
	*Myotis nigricans*	*Bioccala*	*deanei*	(Garnahm, Lainson *et al*., 1971) **	F	RES

Pteropodidae	*Cynopterus sphinx*	*Hepatocystis*	*garnhami*	Landau, 1981	V	HEP
	*Dobsonia moluccensis*	*Hepatocystis*	*pteropi manwelli*	Garnahm, 1966	V	HEP
	*Epomops franqueti*	*Hepatocystis*	*brosseti*	Miltgen, Landau *et al.*, 1977	V	HEP
	*Epomops franqueti*	*Hepatocystis*	*epomophori*	(Rodhain, 1926)	V	HEP
	*Hypsignatus monstrosus*	*Hepatocystis*	*carpenteri*	Miltgen, Landau *et al.*, 1980	V	HEP
	*Lyssonycteris smithi*	*Plasmodium*	*voltaicum*	Van der Kaay, 1964	V	HEP
	*Myonycteris torquata*	*Hepatocystis*	*perronae*	Landau and Adam, 1971	V	HEP
	*Pteropus alecto*	*Johnsprentia*	*copemani*	Landau, Chavatte *et al.*, 2012	V	RES
	*Pteropus alecto*	*Sprattiella*	*alecto*	Landau, Chavatte *et al.*, 2012	V	RES
	*Pteropus alecto*	*Hepatocystis*	*levinei*	Landau, Humpherey-Smith *et al*., 1985	V	HEP
	*Pteropus gouldii*	*Hepatocystis*	*pteropi*	(Breinl, 1913)	V	HEP
	*Pteropus poliocephalus*	*Hepatocystis*	*levinei*	Landau, Humpherey-Smith *et al*., 1985	V	HEP
	*Roussetus leachi*	*Plasmodium*	*rousseti*	Van Riel, Herniaux-L’Hoëst *et al.*, 1951	V	HEP

Hipposideridae	*Hipposideros armiger*	*Hepatocystis*	sp.	Manwell & Kuntz, 1966	V	HEP
	*Hipposideros bicolor*	*Bioccala*	sp.	Eyles, Dunn *et al.*, 1962	F	RES*
	*Hipposideros cyclops*	*Dionisia*	*bunoi*	Landau, Chabaud *et al.*, 1980b	♀F ♂V	RES*
	*Hipposideros cyclops*	*Plasmodium*	*cyclopsi*	Landau & Chabaud, 1978	M	HEP
	*Hipposideros galeritus*	*Hepatocystis*	*bainae*	Mialhe & Landau, 1977	V	HEP
	*Hipposideros galeritus*	*Hepatocystis*	*rodhaini*	Landau, Miltgen *et al.*, 1976b	V	HEP
	*Hipposideros larvatus*	*Biguetiella*	*minuta*	Landau, Baccam *et al.*, 1984	M	HEP
	*Hipposideros larvatus*	*Hepatocystis*	sp.	Duval, Robert *et al.*, 2007	V	HEP*
	*Hipposideros larvatus*	*Nycteria*	*brucechwatti*	Landau, Baccam *et al.*, 1984	M	RES

Rhinolophidae	*Rhinolophus hildebrandti*	*Nycteria*	*congolensis*	(Krampitz & Anciaux de Faveaux, 1960)	M	HEP
	*Rhinolophus sylvestris*	*Nycteria*	*gabonensis*	Rosin, Landau *et al.*, 1978	M	HEP
	*Rhinolophus* sp.	*Nycteria*	*krampitzi*	Rosin, Landau *et al.*, 1978	M	HEP

Type of gametocytes: M = “*malariae*”, F = “*falciparum*” and V = “*vivax*”; site of schizogony: HEP = hepatocyte and RES = reticulo-endothelial system.

(*) indicate the potential location of schizogony which is unknown;

(**) redescribed from *Eptesicus fuscus* by Marinkelle (1995) who change the genus name *Polychromophilus* by *Bioccala*. The reference of the authors of each taxa are listed in the references.

**Table III.** Key for the identification of the Haemoproteidae of Chiroptera.

Characteristic			Haemoproteidae
1–(4)	Elongated or ellipsoid gametocytes type F		
2–(3)	RES: small schizonts spread through the organism ...................................................................................... (Miniopteridae, Vespertilionidae)		*Bioccala*
3–(2)	HEP: small schizonts in the liver .................................................................................................................... (Hipposiderosidae)		*Biguetiella*
4–(1)	Roundish gametocytes		
5–(10)	Gametocytes type M		
6–(9)	RES		
7–(8)	RES: large schizonts spread in different organs ............................................................................................ (Miniopteridae, Vespertilionidae)		*Polychromophilus*
8–(7)	RES: small schizonts, in the vessels of the liver ............................................................................................ (*Hipposideros cyclops*)		*Dionisia*
9–(6)	HEP: large schizonts in the hepatocytes ........................................................................................................ (Nycteridae, Rhinolophidae)		*Nycteria*
10–(5)	Gametocytes type V		
11–(14)	RES		
12–(13)	RES: large schizonts in the lungs ................................................................................................................... (Pteropodidae)		*Johnsprentia*
13–(12)	RES: small schizonts, in the vessels of the kidney ......................................................................................... (Pteropodidae)		*Sprattiella*
14–(11)	HEP: large schizonts (megaloschizonts) in the liver ....................................................................................... (Pteropodidae, Rhinolophidae)		*Hepatocystis*

M = “*malariae*”, F = “*falciparum*” and V = “*vivax*”. HEP = hepatocyte, and RES = reticulo-endothelial system cells.

### Affinities with the haemospiridians of sauropsids

(Garnahm, 1966), in his treatise of the Haemosporidia, separated them into two distinct lineages, those which multiplied in the reticulo-endothelial system including the haemosporidians of birds and reptiles, and in mammals *Bioccala*, designated at the time as a species of *Polychromophilus*; the others multiplying in hepatocytes, comprising *Nycteria* and *Hepatocystis*. Development in the reticulo-endothelial system is close to the primitive model seen in reptiles and birds and the evolution of schizonts in hepatocytes, is in our view a specialisation.

The gametocytes of the “*malariae*” and “*falciparum*” groups have morphological characteristics in common with parasites of Sauropsidae and are therefore, according to Landau *et al.* (1976a), more archaic than those of the “*vivax*” group. Thus, *Bioccala*, a parasite of vepertilionids and miniopterids is, for us, the genus closest to the primitive species. Its gametocyte is ellipsoidal, more or less elongate, does not fill the erythrocyte, the pigment consists of large, irregularly grouped granules, the nucleus is poorly defined, and an accessory chromatin granule is present; the schizonts are small, invasive and are found in the reticuloendothelial system.

The “*malariae*” group is represented by two different genera: *Polychromophilus* in Vespertilionidae and Miniopteridae, and *Nycteria* in *Nycteris* and *Rhinolophus*. The archaic characters of the gametocytes are the fact that the erythrocyte remains partially visible when the gametocyte is mature, the presence of an accessory chromatin granule and the irregularly grouped, large chromatin granules. However, in contrast to *Bioccala*, the nucleus is well delimited and is rounded. The two genera also differ in their schizonts which are relatively large and non-invasive, in the reticulo-endothelial system for *Polychromophilus* and in hepatocytes for *Nycteris*.

The gametocytes of the “*vivax*” group are homogeneous morphologically and differ from the preceding forms. At maturity, they fill or nearly fill the erythrocyte, lack an accessory chromatin granule, have fine, abundant pigment, dispersed in mature forms, and a distinct boundary between nucleus and cytoplasm. Two species are reticulo-endothelial parasites (*Johnsprentia copemani* and *Sprattiella alecto*), but most of the species known from the Pteropodidae have megaloschizonts, which develop in hepatocytes (*Hepatocystis*)

The chromatin in the nucleus of some *Hepatocystis* species instead of being diffuse is aggregated in the center of a sometimes large clear zone (nucleus “en cocarde”). Otherwise they conform to the other species of the group.

### Molecular data and host spectrum

Molecular data from the different groups of animal Haemosporidia are fragmentary and sometimes contradictory for several reasons: polyparasitism of the natural hosts, imprecise identifications... However, some recent molecular data on bat Haemosporidia (Megali *et al.*, 2011) appear consistent with the morphology and the host spectrum: *Polychromophilus* (= *P. melanipherus*) and *Bioccalla* (= *P. murinus*) form two distinct but close taxonomic groups associated with the Vespertilionidae. The *Hepatocystis* of Peropodidae, in this same work, appear as a sister group of both the two previous groups.

Teeling *et al.* (2005) and Simmons (2005) have proposed, using molecular techniques, a phylogenetic tree and dates for the appearance of the different genera of bats. The hosts of haemosporidians of bats belong to five families:

- The Nycteridae harbour parasites of the “*malariae*” group. They separated from other Emballonuroidea 58-47 mya.
- The Miniopteridae and Vespertilionidae harbour a pair of cosmopolitan parasite genera, *Bioccala* and *Polychromophilus*, often in the same individual, which belong respectively to the “*falciparum*” and “*malariae*” groups. These two families are also ancient and separated according to (Miller-Butterworth *et al.* 2007) 48-39 mya.
- The Rhinolophidae, where *Rhinolophus* harbours species of the “*malariae*” group and *Hipposideros* harbours species of three groups, “*vivax*”, “*malariae*” and “*falciparum*”. They diversified more recently, 39 mya.
- The Pteropodidae, which harbour only species of the “vivax” group are even more recent and diverged from the preceding families 24 mya.

## Conclusion

**Fig. 31.**
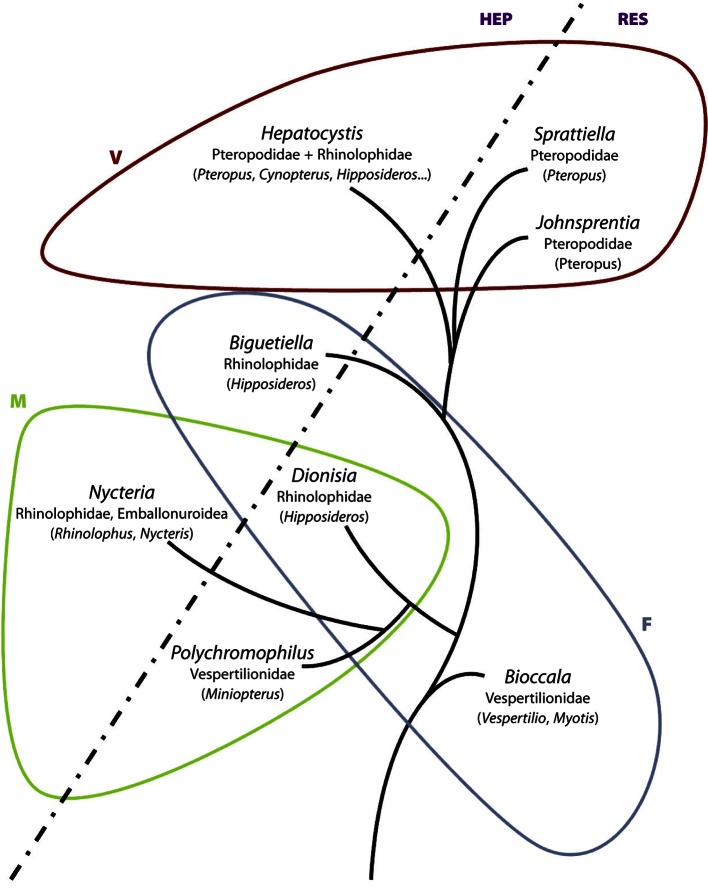
Hypothetical line of ascent of haemoproteid parasites of bats. The oblique doted line separates species with schizonts in the reticuloendothelial system (RES) on the right and the species with schizonts in hepatocytes (HEP) on the left. The green line (low left) encircles parasites with gametocytes of the “*malariae*” type, the blue line (low right) gametocytes of the “*falciparum*” type and the red line (top) gametocytes of the “*vivax*” type

In conclusion, these results appear to be compatible with our hypotheses: the microchiropterans which are, for the most part, older than the megachiropterans, harbour with a few exceptions (in *Hipposideros*) parasites of the “*falciparum*” and “*malariae*” types, which we consider to be the most archaic, while megachiropterans harbour parasites of the “*vivax*” group. A hypothetical line of ascent of haemoproteid parasites of bats synthesising these results is provided in Fig. 31.

## References

[R1] Breinl A. Report of the year 1911. Australian Institute of Tropical Medicine, Townsville, 1911

[R2] Dionisi A. La malaria di alcune specie di pipistrelli. Atti della Società per gli Studi della Malaria, 1899, 1, 133–175

[R3] Duval L., Robert V., Csorba G., Hassanin A., Randrianarivelojosia M., Walston J., Nhim T., Goodman S.M. & Ariey F. Multiple host-switching of Haemosporidia parasites in bats. Malaria Journal, 2007, 6, 157 18045505 10.1186/1475-2875-6-157 PMC2212651

[R4] Eyles D.E., Dunn F.L. & Liat L.B. Blood parasites of Malayan bats. Medical Journal of Malaya, 1962, 17, 87–88

[R5] Garnahm P.C.C. Malaria parasites and other Haemosporidia. Blackwell, Oxford, 1966, 1114 p.

[R6] Garnahm P.C.C. & Heisch R.B. On a new blood parasite of insectivorous bats. Transaction of the Royal Society of Tropical Medicine and Hygiene, 1953, 47, 357–363 10.1016/s0035-9203(53)80016-7 13102576

[R7] Garnahm P.C.C., Lainson R. & Shaw J.J. A contribution to the study of the haematozoon parasites of the bats. A new mammalian haemoproteid, *Polychromophilus deanei* n. sp. Memórias do Instituto Oswaldo Cruz, 1971, 69, 119–125 4997955 10.1590/s0074-02761971000100009

[R8] Krampitz H.E. & Anciaux De Faveaux F.M. Über einiger Haemosporidien aus Fledermaüsen der Höhlen des Berglandes Katanga. Zeitschrift für Tropenmedizin und Parasitologie, 1960, 11, 391–400 13753953

[R9] Landau I. & Adam J.P. Description de schizontes de rechute chez un nouvel Haemoproteidae, *Hepatocystis perronae* n. sp., parasite de Megachiroptères africains. Cahier ORSTOM., Série Entomologie Médicale et Parasitologie, 1971, 9, 373–378

[R10] Landau I., Miltgen F. & Chabaud A.G. Les différents types de gamétocytes chez les hémosporidies de mammifères. Corrélations avec la morphologie des schizontes tissulaires. Hypothèses sur l’évolution du groupe. Annales de Parasitologie Humaine et Comparée, 1976a, 51, 175–187 823859

[R11] Landau I., Miltgen F., Le Bail O. & Yap L.F. *Hepatocystis* de Malaisie IV. Description d’*Hepatocystis rodhaini* n. sp. parasite de Microchiroptères. Annales de Parasitologie Humaine et Comparée, 1976b, 51, 303–307 825011

[R12] Landau I. & Chabaud A.G. Description de *Plasmodium cyclopsi* n. sp. parasite du microchiroptère *Hipposideros cyclops* à Makokou (Gabon), Annales de Parasitologie Humaine et Comparée, 1978, 53, 247–253 697287 10.1051/parasite/1978533247

[R13] Landau I., Rosin G., Miltgen F., Hugot J.P., Leger N., Beveridge I. & Baccam D. Sur le genre *Polychromophilus* (Haemoproteidae, parasite de microchiroptères). Annales de Parasitologie Humaine et Comparée, 1980a, 55, 13–32

[R14] Landau I., Chabaud A.G., Miltgen F. & Baccam D. *Dionisia bunoi* n. gen., n. sp. Haemoproteidae parasite du microchiroptère *Hipposideros cyclops* au Gabon. Annales de Parasitologie Humaine et Comparée, 1980b, 55, 271–280 6773461 10.1051/parasite/1980553271

[R15] Landau I. Description d’*Hepatocystis garnhami* n. sp., parasite du chiroptère *Cynopterus sphinx* (Vahl) en Thaïlande, *in*: Parasitological topics. A presentation volume to P.C.C. Garnham, F.R.S., on the occasion of his 80th Birthday, 1981. Canning E.U. (ed.), Society of Protozoologists, Allen Press, Inc., Lawrence, Kansas, 1981 Special Publication No.1, 166–169

[R16] Landau I., Baccam D., Ratanaworabhan N., Yenbutra S., Boulard Y. & Chabaud A.G. Nouveaux Haemoproteidae parasites de Chiroptères en Thaïlande. Annales de Parasitologie Humaine et Comparée, 1984, 59, 437–447 6439099

[R17] Landau I., Humpherey-Smith I., Chabaud A.G., Miltgen F., Copeman B. & Boulard Y. Description et transmission expérimentale de l’haemoproteidé *Hepatocystis levinei* n. sp. Annales de Parasitologie Humaine et Comparée, 1985, 60, 373–382

[R18] Landau I., Chavatte J.M. & Beveridge I. *Johnsprentia copemani* gen. nov., sp. nov. (Haemoproteidae), a parasite of the flying-fox, *Pteropus alecto* (Pteropodidae), from Queensland. Memoirs of the Queensland Museum, 2012, 56, 61–66

[R19] Manwell R. & Kuntz R.E. *Hepatocystis* of Formosan Mammals with description of a new species. Journal of Protozoology, 1966, 13, 670–672

[R20] Marinkelle C.J. The haemoproteid parasite, *Bioccala deanei*, from a Colombian bat, *Eptesicus fuscus* (Vespertilionidae). Annals of Tropical Medicine and Parasitology, 1995, 89, 89–91 7741601 10.1080/00034983.1995.11812935

[R21] Megali A., Yannic G. & Christe P. Disease in the dark: molecular characterization of *Polychromophilus murinus* in temperate zone bats revealed a worldwide distribution of this malaria-like disease. Molecular Ecology, 2011, 20, 1039–1048 21073585 10.1111/j.1365-294X.2010.04905.x

[R22] Mialhe E. & Landau I. Description d’*Hepatocystis bainae* n. sp. (Haemoproteidae) parasite d’*Hipposideros galeritus*, Microchiroptère de Malaisie. Annales de Parasitologie Humaine et Comparée, 1977, 52, 385–390 412454

[R23] Miller-Butterwoth C.M., Murphy W.J., O’Brien S.J., Jacobs D.S., Springer M.S. & Teeling E.C. A family matter: conclusive resolution of the taxonomic position of the long-fingered bats, *Miniopterus*. Molecular Biology and Evolution, 2007, 24, 1553–1561 17449895 10.1093/molbev/msm076

[R24] Miltgen F., Landau I., Rosin G. & Erard C. *Hepatocystis brosseti* n. sp., Haemoproteidae parasite d’*Epomops franqueti*, Pteropinae, au Gabon. Annales de Parasitologie Humaine et Comparée, 1977, 52, 589–596 418727 10.1051/parasite/1977526589

[R25] Miltgen F., Landau I. & Bradbury J. *Hepatocystis* d’*Hypsignathus monstrosus* (Pteropinae) au Gabon. Description d’*Hepatocystis carpenteri* n. sp. Annales de Parasitologie Humaine et Comparée, 1980, 485, 55–490 6784655

[R26] Rodhain J. *Plasmodium epomophori* n. sp., parasite commun des roussettes épaulières au Congo Belge. Bulletin de la Société de Pathologie Exotique, 1926, 8, 726–729

[R27] Rosin G., Landau I. & Hugot J.P. Considérations sur le genre *Nycteria* (Haemoproteidae) parasite de Microchiroptères africains avec description de 4 espèces nouvelles. Annales de Parasitologie Humaine et Comparée, 1978, 53, 447–459 105663

[R28] Simmons N.B., An Eocene big bang for bats, Science, 2005, 307, 527–528 15681371 10.1126/science.1108871

[R29] Teeling E.C., Spinger M.S., Madsen O., Bates P., O’brien S.J. & Murphy W.J. A molecular phylogeny for bats illuminates biogeography and the fossil record. Science, 2005, 307, 580–584 15681385 10.1126/science.1105113

[R30] Van der Kaay H.J. Description of a new *Plasmodium*: *Plasmodium voltaicum* sp. nov. found in a fruit bat, *Roussetus smithi* in Ghana. Annals of Tropical Medicine and Parasitology, 1964, 58, 261–264 14212880 10.1080/00034983.1964.11686241

[R31] Van Riel J., Herniaux-L’hoest D. & Herniaux-L’hoest J. Description of a *Plasmodium* found in bat, *Roussetus leachi*. Parasitology, 1951, 41, 270–273 14911220 10.1017/s0031182000084109

